# Populism, cyberdemocracy and disinformation: analysis of the social media strategies of the French extreme right in the 2014 and 2019 European elections

**DOI:** 10.1057/s41599-023-01507-2

**Published:** 2023-01-18

**Authors:** Uxía Carral, Jorge Tuñón, Carlos Elías

**Affiliations:** Carlos III University of Madrid, Madrid, Spain

**Keywords:** Politics and international relations, Sociology, Cultural and media studies, Cultural and media studies

## Abstract

Since the European elections in 2014, the populist formations had been increasing the shares of power that their masses finally claimed in the May 2019 elections. In the case of France, this rise of the Rassemblement National has even given rise to a new political regime: the Europeanism of Macron vs. the Euroscepticism of Le Pen. This paper aims to delve into the communication strategy of the latter formation, to see how it has been able to shift the party’s fascist image towards that of a protector of national sovereignty and interests (against the European ones). To this end, the 1256 tweets posted on its official Twitter profile during the fortnight of the 2014 and 2019 European election campaigns have been qualitatively analysed. The results point to a whitening of its image, in order to present itself as a potential voting option for the majority of the French electorate while retaining a certain populist essence.

## Introduction

The rise of populism, in the light of the examples of the election of Donald Trump and the result of the Brexit referendum, has become one of the greatest European concerns in the so-called post-truth or disinformation era (Tuñón et al., 2019). It is precisely at the European level that populist organisations have best positioned social networks as the centre of their communication and public relations strategy. In fact, in order to stop being a minority and increase their support base, they have become aware of the need to plan transversal organisational communication strategies that are necessarily anchored in social networks (Schoenebor, 2011). An example of this is the Rassemblement National (RN), an extreme right-wing party led by Marine Le Pen, whose popularity among the French electorate reached an all-time high in the last elections. After its resounding victory in 2014, the party managed to regain the honorary title of France’s leading political force in the last (2019) European elections, even beating the centrist option Renaissance, the party of the French president and visible head of the current EU, Emmanuel Macron (Habermas, 2017; Bouza and Tuñón, 2018). This success is mainly due to the change of image and story that the political organisation has managed to show to audiences through an evolution in its communication plan. Specifically, the training—given the intrinsic characteristic of its qualification as a populist—stands out for its good use of social networks. For this reason, its discursive practices in the online world constitute the object of study that will occupy this research.

Currently, it is essential for organisations’ public relations to make use of emerging technological methodologies, especially online and through social networks. While several academics have analysed the political discourse on new media and social networks such as Twitter (Eom et al., 2015; Kreiss, 2016; Larsson and Kalsnes, 2014), we transfer this analysis to a small part of the communication action plan of political organisations in election campaigns, taking as a case study the party par excellence of the French extreme right. In fact, at present online formulas are crucial for political and institutional actors who aspire to engage citizens and involve audiences (Campos, 2017; López et al., 2017), either at a supranational level as in the case of EU communication strategies (Papagianneas, 2017; Bouza and Tuñón, 2018; Tuñón, 2017), or at a national level as this paper intends to demonstrate.

In short, this work expects to prove that the RN, as an example of European populism, has known how to adapt its communication strategy to the emergence of social networks, increasing the performance of its Twitter campaign from 2014 to the 2019 European Parliament elections. The need for transversality in the party’s image in the 2019 elections has promoted different political stakeholders’ representation in the official account, unlike what happened 5 years before. Likewise, the level of engagement of the growing audience has increased. As a result, this change in strategy is aimed at whitening the party’s image and creating a false establishment, which will allow it to show itself as just another party within the French political media class. However, the change has not penetrated deeply and the RN remains a national-populist formation that feeds on confrontation and propaganda.

## Populisms and the consolidation of cyberdemocracy in the current Europe

Since the beginning of the current century, we have been witnessing the rebirth of Populism as an essentially political ideology of Western democracies (Mudde, 2004). Although populist ideology dates from the nineteenth century (Mazzoleni, 2014), it has re-emerged owing to the popularity and electoral successes of extreme right-wing populist parties in European countries such as France, Italy and Austria (Mouffe, 2005). And more recently in Hungary, the United Kingdom and Spain (Shein, 2020; Mudde, 2016; Alonso and Casero, 2018).

Anchored in the emergence of mass societies (Taggart, 2000), it was argued from the outset that the mass media were the cause and not just the consequence of the rise of populism (Kornhauser, 1959). In that sense, it is defended that the development of the media industry has provided the ideal context for the growth of populism (Mudde, 2004). In that sense, technological developments coupled with the popularisation of social networks have helped populism to break free from the constraints of traditional media. In fact, it has allowed it to bypass them and communicate directly with citizens, without any intermediation.

For all these reasons, the chameleonic and malleable nature of populist discourse (Taggart, 2000) has found its perfect breeding ground in social networks. In particular, in the context of unmediated communication (Bennett, 2012), populist strategies have been benefiting from real-time data analysis and audience micro-segmentation (Guerrero et al., 2020), as happened in the famous case of Cambridge Analytica.

Since the last decade, political communication has not been able to set aside social networks in general, and Twitter in particular, as intermediary channels for the message. With great success, they have shown that they are useful to project a precise profile of a candidate (Jivkova et al., 2017). Therefore, their influence on the communication framework of political organisations has only grown in all advanced democracies (Bimber, 2014; López et al., 2017). In fact, the communication plans of political organisations can no longer be understood without the use of social networks. These have become essential tools for interaction with younger audiences. In particular, neither academics nor political parties or leaders have been able to resist the potential of the social network best suited to political debate and communication strategies: Twitter.

There is already a considerable amount of research on its practical use by political parties and leaders. It has been analysed its application in the context of election campaigns through different approaches: strategic communication by political organisations and their candidates (Parmelee and Bichard, 2012); campaign information and speech dissemination (Jackson and Lilleker, 2011); promotion of audience participation and mobilisation (Gainous and Wagner, 2014); or promotion, image and self-referral in relation to the campaign itself (Jivkova et al., 2017). Likewise, work has been done on the extension or frequency of its use; on the themes addressed; or on the reasons for its preferential use (Bouza and Tuñón, 2018, p. 1240). Despite this, at the supra-state level, there is some debate about the effect of such networks on the communication of pan-European political organisations (Barisione and Michailidou, 2017).

In view of its ability to reduce transaction costs, researchers such as this one analyse the main functions attributed to Twitter in the context of political discourse of the French far-right populist party RN, namely: its use in the configuration of public relations and communication strategies; the functions attributed/expected to the social network from the political parties; the distribution of the message, its impact and engagement on the audiences; or the authorship and personalisation of the party leaders, themselves.

### Populisms

Although there is no scientific consensus to define populism (Taggart, 2000), which can even operate as an “empty signifier” (Laclau, 2007), certain guidelines common to populist parties have been distinguished, namely: centrality or personalisation in a charismatic leader capable of attracting the masses and facing them to the political or economic elites; demagogic reductionism pretending to offer simple solutions to complex problems; and/or apocalyptic discourses channelled lately through television and social networks.

It has also been verified that the weakening of traditional political parties, the blurring of the left-right ideology in Western democracies (Mouffe, 2005) or the European polycrisis: Euro, Refugees, Brexit and Covid-19 during this century, have created the breeding ground for the rise of populisms.

Part of its success has to do with its ability to shape itself chameleonically to left or right-wing postulates (Taggart, 2000), proposing ideologically less strict and/or combined positions (Mudde, 2004) for post-industrial societies. This is an eminently pragmatic model, in search of hegemony and power. For two of the greatest theorists of populism, Laclau and Mouffe, both its ambiguity and its malleability (Laclau, 2007), as well as the polysemy of its preferred construction “the people”, as the subject and object of different social demands, are inherent in this discourse. Indeed, “the people” can refer to the most disadvantaged social classes or to the nation as a whole, as appropriate. As Müller (2016) agrees, the symbolic representation of the “real people” as opposed to the dominant elites allows populists to deduce the “correct” political position on each occasion.

The malleability of populism means that both left and right-wing ideologies can be integrated into it. As a consequence of the rejection of the political establishment, there are two proposals for the protection of citizens within neo-liberalism and globalisation: right-wing populism addresses these problems from an individual perspective, identifying and blaming those responsible (often minorities); while left-wing populism focuses on the reversal of structural inequalities, as well as on social protection (Fraser, 2017, p. 282).

As for the discourse, the populist is simple, direct and emotionally connoted (Taggart, 2000). It tends towards reductionism (Engesser et al., 2017) and is usually as malleable or adaptable as it is ambiguous. Populist communication in particular focuses on “the people” and their traditional values, as a general framework (Jagers et al., 2007), which generates an imagined community, supposedly homogeneous, confronted with the elites, but excluding the extremes (the minorities).

Both Mouffe (2005) and Mudde (2004) assimilate populism as an archaic form of identification based on emotional belonging to a community, in order to avoid feeling submitted or excluded. In particular, extreme right-wing populism points in its discourses to migration, Islam or the EU itself. This is due to its supposed link with the corrupt elite or the challenge they argue they pose to those who decide what the common values or interests of these imagined collectivities are (Guerrero et al., 2020, p. 3). In short, for Rooduijn (2014), the common factors in the populist discourse around the world are the central position of the “people”, the criticism of the elites, the consideration of the “people” as a homogeneous entity, and the existence of a crisis.

### Populism, cyberdemocracy and social media

Theorists such as Mazzoleni (2007) argue that the media have been precisely the necessary cooperators of populism, giving it visibility. In fact, populist politicians, as we will see in this paper mostly in the case of Marine Le Pen, are usually charismatic and able to adapt their discourses to the demands and typologies of both, the media and their audiences. Their appeal to the emotions and their presentation as “members of the people” in accordance with popular tastes, makes them indispensable to the media themselves.

Although their behaviour has been analysed to date in different formats such as the written press (Rooduijn, 2014) or television (Jagers and Walgrave, 2007); we would like to refer specifically to their interaction in social networks. In fact, they have not hesitated to incorporate this tool intensively into their most recent communication strategies. This has fostered more pronounced rhetoric to attack the elites (Klinger and Svensson, 2015); as well as greater emotionality and personalisation (Enli, 2017; Falkner and Plattner, 2020; Gattermann, 2020).

Indeed, not only have social networks come to play a decisive role in the communication strategies of populist political parties, but the dependence of the conventional media on them has led to a change in the domain of communication that Lévy (2004) has called ‘cyberdemocracy’. At that time, it was understood as the transposition of real democracy into the digital public sphere with the aim of conquering “a new type of transparent State at the service of the collective conscience” (p. 11). The idea was rooted in “an autonomous space that acted as a place for forming groups, debate, co-decision making and a laboratory for experimenting with new forms of deliberative democracy” (Castells, 2016, p. 144).

However, in 2016 Sénit, Kalfagianni and Biermann updated the term of cyberdemocracy more specifically by defining it as “the democratisation of decision-making processes through the use of ICT” (p. 534). The authors wanted to highlight the use of social media as a decisive tool to enhance people’s sovereignty in the modern political scope since it had been a key factor in the success of multiple democratic processes such as Obama’s 2008 presidential campaign in the USA, the citizen movement of 15-M in Spain or the Arab Spring in the Middle East (Christensen, 2011).

Despite giving this credit, Sénit et al. (2016) still doubted the power of cyberdemocracy to include, in the course of political decision making, the whole of civil society participation through the different social media channels. It is true that, unlike the offline scenario, it is the connected society that has the power to grant a long or ephemeral path to political becoming (Mosca and Vaccari, 2011). Indeed, the emergence of Information and Communication Technologies (ICTs) has given the user community the power to decide on the prosperity, popularity and visibility of political conversation (Mazzoleni, 2007).

However, notable figures in social media such as politicians still have control of the conversation over their followers. They benefit from disintermediation (Aalberg and De-Vreese, 2016) and hold preponderance enough to choose which topics to put on the agenda and which to leave out (agenda setting), especially populist leaders who tend to put controversial themes on the table. Moreover, they can even filter information or disseminate opinions contrary to what has been said in the media. Thus, the omnipresence of Twitter in the daily agenda has created a need for organisations to be present and to update themselves. So that their digital behaviour already has an impact on their actions and dialectic patterns which are maintained in real life (Elías, 2015).

Therefore, cyberdemocracy or the use of social media in the political scope is implying a restructuring of the political communication system (Chadwick, 2006). Furthermore, the own adaptation of these actors’ communication strategies to this medium indicates its relevance, especially during the planning and campaign periods (Maarek, 2011; Tuñón and Catalán, 2020).

Specifically, in the case of the social network Twitter, it has been verified active and regular management of its accounts favours the dynamisation of dialogue, the dissemination of the message and the increase in popularity of the political profile (Larsson, 2015), something of vital importance for organisations of a populist nature. Moreover, its interactivity through mentions and hashtags gives rise to threads of conversation that, indirectly, promote “the creation of links between the various users” (Larsson, 2015, p. 89), from which circles of support can end up being developed. Likewise, participation—in the form of retweets (RTs), ‘I like you’ (favs) and comments—helps to reinforce attention to the organisation’s account, giving it greater visibility, so that it translates into an increase in followers and, therefore, greater influence.

In fact, the use of Twitter has been most noted: (a) as a mere channel of maximised self-promotion during election campaign times (Jungherr, 2014); (b) to mobilise users and encourage them to participate (Gainous and Wagner, 2014); and (c) as a hook to capture the attention of the mainstream media and focus coverage on them or influence their publications, by complaining or promoting their interventions in their networks (Stromer-Galley, 2014).

## Study case, methods and research questions

We intend to apply the barely explained theoretical context to the case study of the RN, as an example of the success of the French populist extreme right (Delwit, 2012; Stéphane and Olivier, 2015). A party that has been on the rise for the past two decades for three reasons: (a) the inability of the French left to combat the arrival of the radical populist phenomenon; (b) the use of the fragile and fragmented European space and its choices as springboards for making its Europhobic discourse visible; and (c) its own change in communication strategy as a result of the metamorphosis undergone by the transition between the leadership of founder Jean Marie Le Pen and that of his daughter Marine.

Although the signs of identity of the party have remained unchanged, namely: connection with the citizenry through charismatic leaders; discourse challenging the existing order; or integration into it of the needs of security, social cohesion and identity of the electorate (Fernández-Vázquez, 2019, p. 32); it has been argued that the party’s postulates have been de-ideologized in favour of a pragmatic broadening of the party’s electoral base, within the discourse of Marine Le Pen in relation to the orthodoxy of her father, Jean Marie.

Indeed, the aim of this research is to demonstrate a variation in the way in which the RN relates publicly through Twitter, currently considered as an element with “power and value” within “public relations” and “mass self-communication” (Castells, 2009, p. 68). The demonstration of this purpose would add further evidence to the accumulation of those described by various authors (Fernández-Vázquez, 2019; Alduy, 2017; Betz, 2017, Crépon et al., 2015, or Albertini and Doucet, 2016) in order to affirm that there is a whitening of the image of the RN since the arrival of Marine Le Pen in 2011.

Therefore, the main hypothesis of our work involves that the RN’s online communication strategy has evolved in order to widen its potential range of voters. To this end, the first aim is to demonstrate the consolidation of Twitter as a communication tool for political organisations; and the second, is to relate the increase in the number of authors to the engagement in their messages. The achievement of these two objectives would demonstrate an emerging trend in the organisational communication of political parties that Eom et al. (2015) explain as the modelling of the dynamics of collective attention/participation by users in social networks. Therefore, the research questions are the following:

1. Does RN value its presence on Twitter as a main resource of its communication strategy?
2. Has the openness of its Twitter posts’ authorship meant a greater virality of its message?
3. Can the RN still be considered a populist party or has it already adapted to the French establishment?

On the one hand, with regard to the period of time analysed, the aim was to make a comparison with the peak of the party after its regeneration, which meant studying the RN after the appointment of Marine Le Pen as a leader (16/1/2011). Furthermore, it is necessary to bear in mind the characteristics of France’s electoral system, which consists of two rounds. This factor prevents equal treatment of data between all elections. Consequently, screening the twelve times that the French has gone to the polls since 2011, the perfectly comparable results with the best percentage for the RN were given in the European Parliament (EP) elections. The study was therefore framed around the two EP election campaigns of 2014 and 2019 (9-24/5/2014 and 10-25/5/2019).

In view of this pluralistic space of conversation, it was decided to systematise the scenario by using a hybrid quantitative methodology composed of computational and manual procedures. Content analysis is one of the most useful methods for the study of electoral campaigns, since it is an objective technique that allows for the description, but also the interpretation and generalisation of patterns. Thus, the corpus is made up of 1256 tweets issued by the official profile of the RN (@RNational_off), of which 254 publications correspond to the year 2014, and 1002 to the period 2019.

With the Tweets application, free and online, the tweets have been compiled and the analysis carried out, for which an own elaboration sheet has been used (Table 1 ANNEX), inspired by previous research (Congosto, 2015; Calvo et al., 2017; Marcos, 2018). Thus, a system of eight variables was developed, arranged in three levels. In this sense, it should be stressed that it has been necessary to establish ‘pro rata intervals’ following the recommendations of previous research on engagement (Giglietto and Selva, 2014). This decision is due to the disparity of results induced in a first review prior to the beginning of the analysis and the desire to avoid possible deviations and biases. Subsequently, given the complexity that would be involved in manually calculating such an amount of tweets, the coded data were entered into the database of the IBM SPSS programme (version 25), with which we were able to cross the variables to obtain the percentages and correlations that make up the core of this content analysis and which are presented below.

**Table 1:** Contents analysis sheet/own elaboration.

**Storytelling level**		
1. Subjects *about what?*	2. Autorship *who tweets?*	3. Purpose *with which aim?*
1. Immigration/security/borders	1. Own profile of RN/FN	1. Propaganda
2. Foreign affairs (EU & Brexit)	2. Marine Le Pen	2. Programme info
3. Economy/work/energy	3. Jordan Bardella	3. Promotion in media
4. Social policies (education, health, euthanasia, feminism)	4. RN members or allies	4. Confrontation to media and politicians
5. Justice	5. Media	5. Complaint
6. Electoral campaign/party	6. Rivals	6. Vote
7. Others (climate, agriculture, culture, identity)	7. Others	7. Emotional message and patriotism
**Interaction level**		
4. Participation *to whom is addressed the tweet?*	5. Content *which is the main element?*	
1. Tweet without mention	1. Link to own web/social networks	
2. Tweet with @# mention to a RN member	2. Media links	
3. Tweet with @# mention to a rival	3. Picture	
4. Tweet with @# mention to a media channel	4. Video/audio	
5. Tweet with @# mention to others (institutions, etc.)	5. Text (or another tweet)	
**Engagement level**		
6. Sharing *how many RTs?*	7. Likes *how many favs?*	8. Comments *how many responses?*
1. 2–5	1. 2–5	1. 2–5
2. 6–25	2. 6–25	2. 6–25
3. 26–100	3. 26–100	3. 26–100
4. 101–200	4. 101 – 200	4. 101–200
5. 201–300	5. 201–300	5. 201–300
6. 301–400	6. 301–400	6. 301–400
7. 401–500	7. 401–500	7. 401–500
8. More than 500 RTs	8. More than 500	8. More than 500
9. Zero	9. Zero	9. Zero

## Results and discussion

With the aim of answering the three questions raised before, in this article we did compare the behaviour of RN framed to each electoral campaign from various perspectives based on three hypotheses:

### Hypothesis I: The RN value its presence on Twitter as a main resource of its communication strategy

From a simple first view, we observe a significative change in the RN strategy: an increment of 400% in the number of tweets published or retweeted by the own political party on its official Twitter account, moving from 254 messages in 2014 to 1.002 during the 2019 campaigns. However, RN has not only tweeted more but also did better.

The change in the origin of audiovisual content signals the first major transformation in the strategy carried out in these 5 years. In 2014, 98% of the links directed the user to the website, because channelling the message through a single communication channel reduced the risk of distorting the story. However, the handling of social networks was very rough and monotonous, and the appearance of the publications was unattractive. Communication was in one direction only (party to followers) and the exercise was uncomfortable for the user, as the style of publication forced readers to migrate from the social network to the web in order to continue deepening the content. Therefore, all this led to a very low or sometimes no engagement of the audience, which caused a positive reaction in relation to the handling of the networks in subsequent election campaigns.

**Fig. 1:**
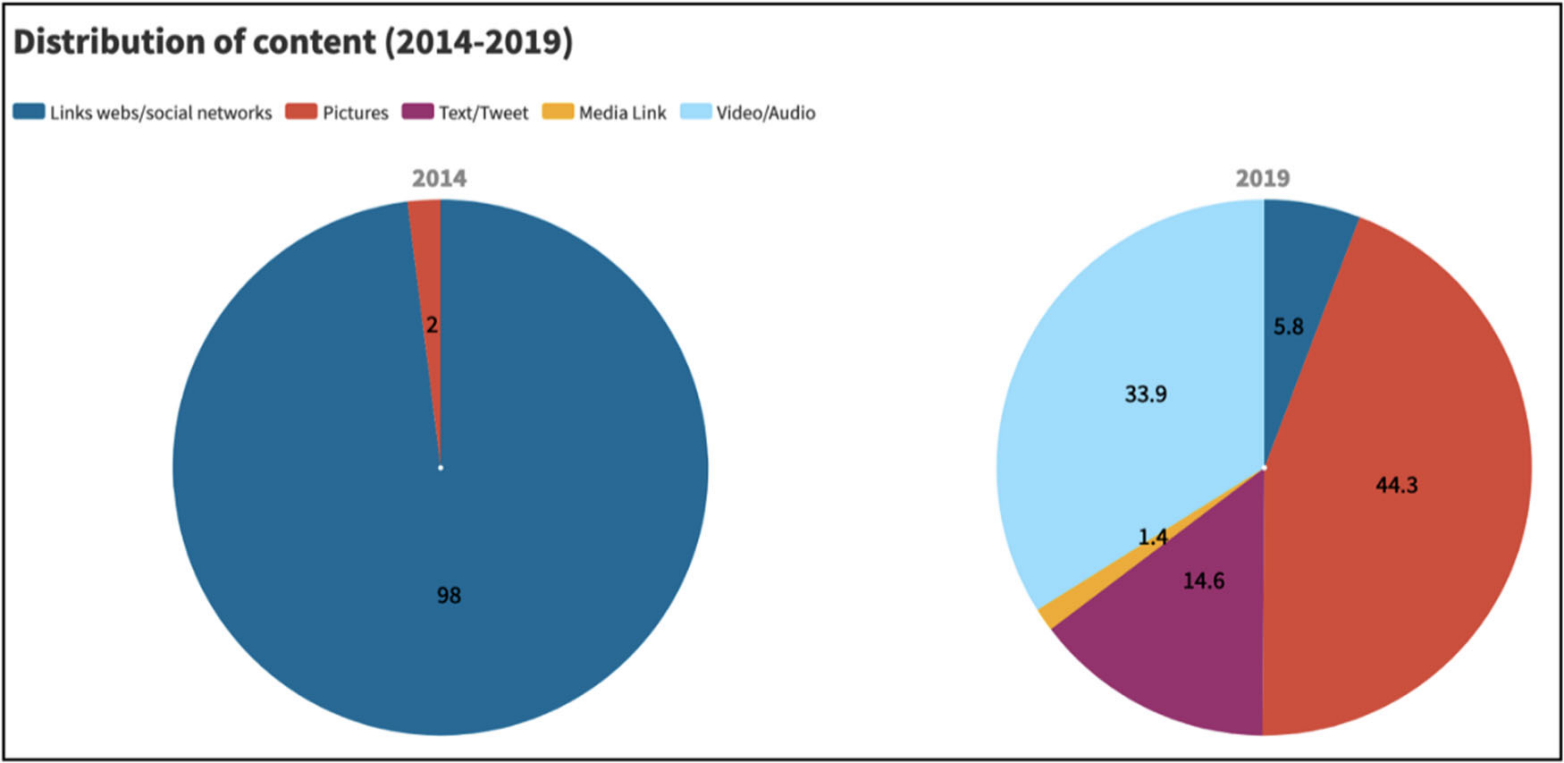
Distribution (%) of the content of the RN tweets (2014–2019)/Own elaboration.

Firstly, we have realised that the content inserted in the tweets causes an increase in the potential expansion of his message, and therefore would help to increase the circles of support. In fact, the 2019 election campaign has had a greater dynamism in communication, reflected in the fact that 86.8% of the tweet sample was accompanied by images (44.3%) and videos or audio (33.9%), mainly (Fig. 1).

Secondly, another evidence of its variation when it comes to communication has been given in the tweets published by the RN with direct links to news coming from the media’s own website. Given the little or no media focus on the far-right party in 2014, the relationship in 2019 has partially changed. The populist party does not intend to seek allies in the media, but it is aware that it needs to be present in the media. For this reason, small video clips of its interventions in radio and television programmes have become widespread, with one in three tweets published being directed at this future or past promotion of the different members in the media. Likewise, RN has timidly opened the door, and paradoxically through social networks, to a closer relationship with traditional media.

Although it is only equivalent to 14 tweets out of a total of 1002, it should be read in greater depth. The RN’s progress in spreading its message has been galloping in recent years. It has gone from 2014, when it was feared that its message would be contaminated if it were shown on its own profile, to 2019, with more than 50 messages a day, with audiovisual content, and even encouraging its followers to read what the media, which in part are its rivals, say about them. It must also be admitted, however, that the shared links were chosen because they fit in with the RN’s story for the European elections.

Third and finally, the emergence of small talks, which were completely cancelled in 2014, in these past elections, have been motivated by two techniques. One of these is to insert the previous tweet next to the text of the reply (14.6% of the sample in 2019) or to mention it in the tweet itself published by the French party. In this sense, 5 years before, messages without appeals constituted 80% of the NR’s communication model, whereas in 2019 they only covered a third of the publications.

However, although it may seem that the RN has chosen to introduce two-way communication into its strategy, at no time has it created a Twitter thread with another political actor. The mentions are just provocative bait that is exposed in front of the audience because it is only interested in making its followers aware of the figure of its enemies (‘the others’) according to the description in 140 characters that benefits the RN.

### Hypothesis II: The openness of authorship meant a greater virality of his message

The evolution of the RN in social networks is dictated by a need for progression at the political level. Le Pen’s party achieved the best results in its history in the European elections in 2014 and was at the top of the ranking of political forces in France for the first time. Therefore, the objective for the 2019 elections was to widen the gap with the other formations, or, in the worst case, not to lose that first position.

Changing its name to *‘Rassemblement National’* implies the obligation of greater pluralism. This does not mean, however, that their leader loses supreme power and that the hierarchical positions of the RN team are blurred; rather, it is a question of visibility. Therefore, in 2019, the first gesture that expressed this new position of the RN was the expansion in the number of voices authorised to proclaim the party’s message, by giving them special visibility in the official profile.

**Fig. 2:**
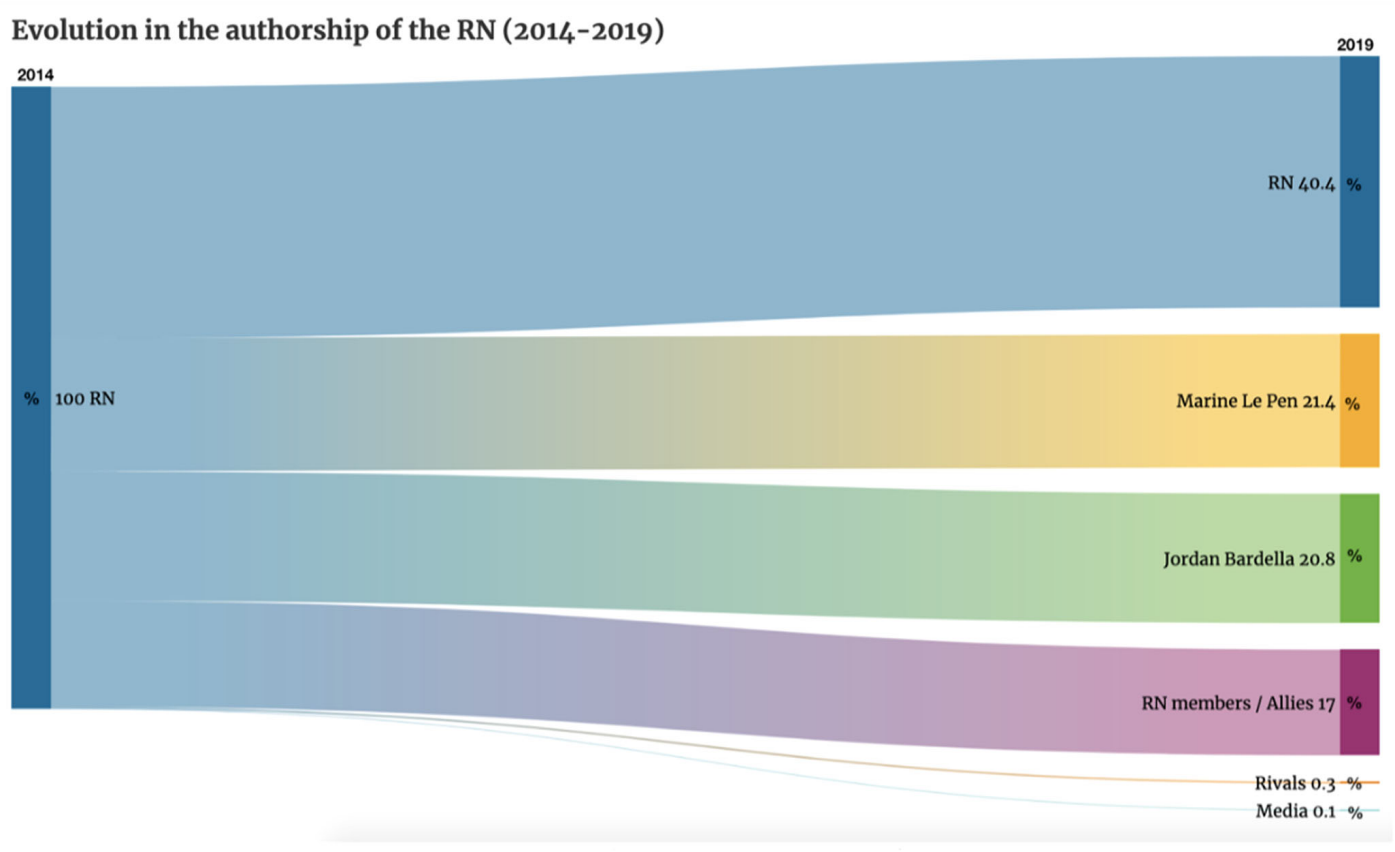
Evolution in the authorship of the RN (2014–2019)/Own elaboration.

Thus, Fig. 2 shows the difference between the 2014 electoral period, where all of the publications issued came from @RNational_off itself, compared to only 40% of messages written by the account in 2019. This disparity means that six out of ten tweets on the RN wall are retweets to other party members. Among these, the first figure to be given space is undoubtedly the president, Marine Le Pen (21.4%). Next up is Jordan Bardella (20.8%), since, in a European campaign, the head of the list must function as a leader. From then on, the weight of the hierarchy is imposed, and the rest of the party members have a lower degree of visibility.

As a result, the message received by RN supporters comes from many different personalities, widening the possibilities of connecting with various user prototypes and seeking their vote from the position with which they are most recognised (the president, the European leader, the local mayor, etc.). On the other hand, there is hardly any room for agents who could misrepresent their message. Only 0.3% of the sample of 1002 tweets collected in 2019 came from political rivals and 0.1% were retweets to the media.

**Fig. 3:**
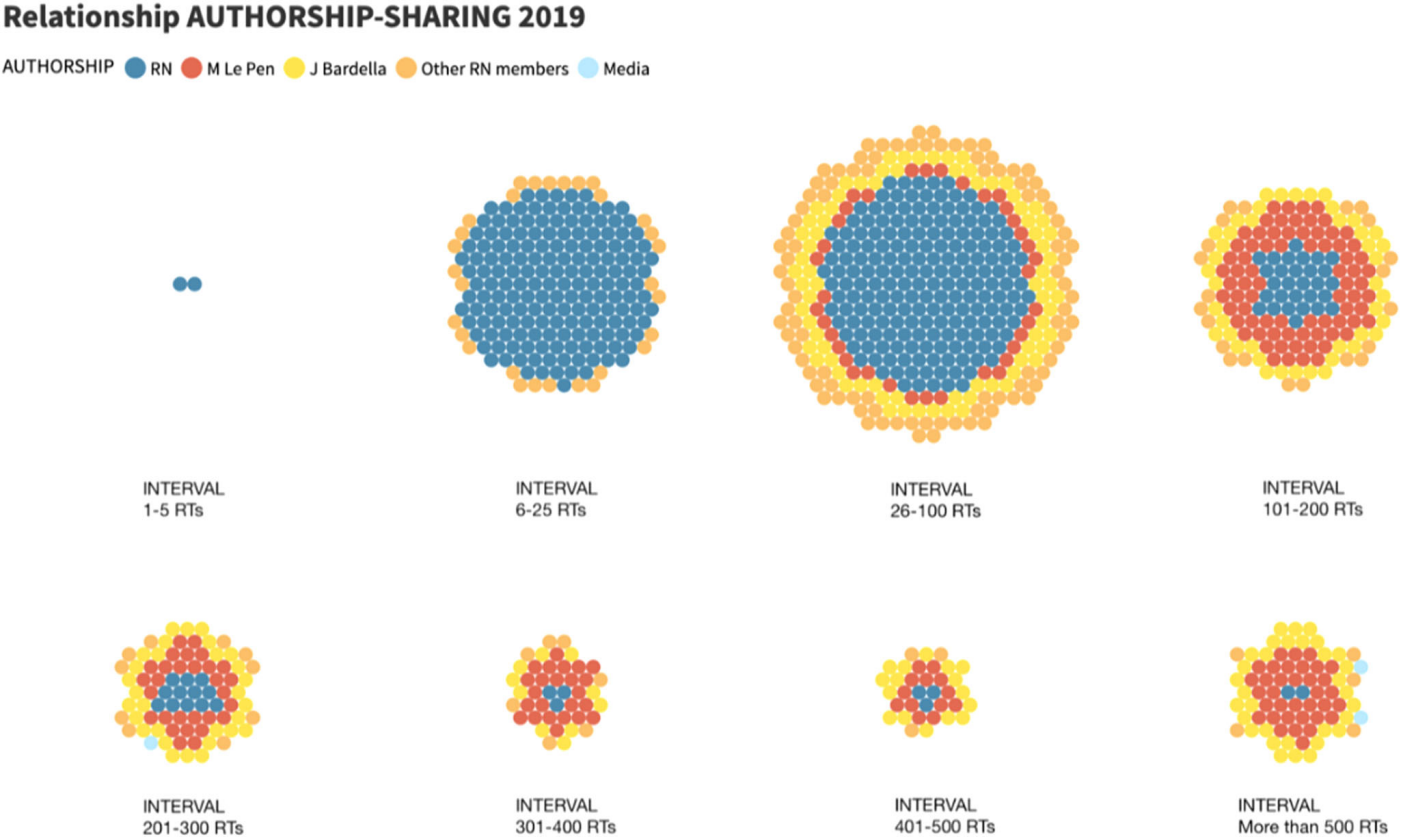
Relationship between RN authorship and radio range in RTs/Own elaboration.

An example of the RN’s new strategy of weaving a story through a multiplicity of actors is the level of engagement compared to 2014 and 2019. In 2014, when the authorship of tweets remained entirely under the guidelines of the official account, the most frequent average of RTs (in 57.9% of cases) was in the range of 6–25 retweets of RN messages by followers. On the other hand, after the distribution of the public space of the RN account for 2019, the link of the RN followers with the tweets has been strengthened, since users usually retweet 26–100 times the messages—as can be seen in the third interval of Fig. 3.

Moreover, there is a turning point from the fourth interval (101–200 Rts). The published tweets—each circle equals one tweet—by the party constitute a larger part of the sample, but they achieve a lower range in number of retweets, predominating only in the first three ranges. On the other hand, those messages published by the other authors, and retweeted by the RN profile, make up a smaller part of the sample, but have, in the upper intervals, a higher range than those of the account itself. This can be seen in the case of Marine Le Pen and Jordan Bardella, whose messages are more likely to receive more than 500 Rts than those of the RN.

**Fig. 4:**
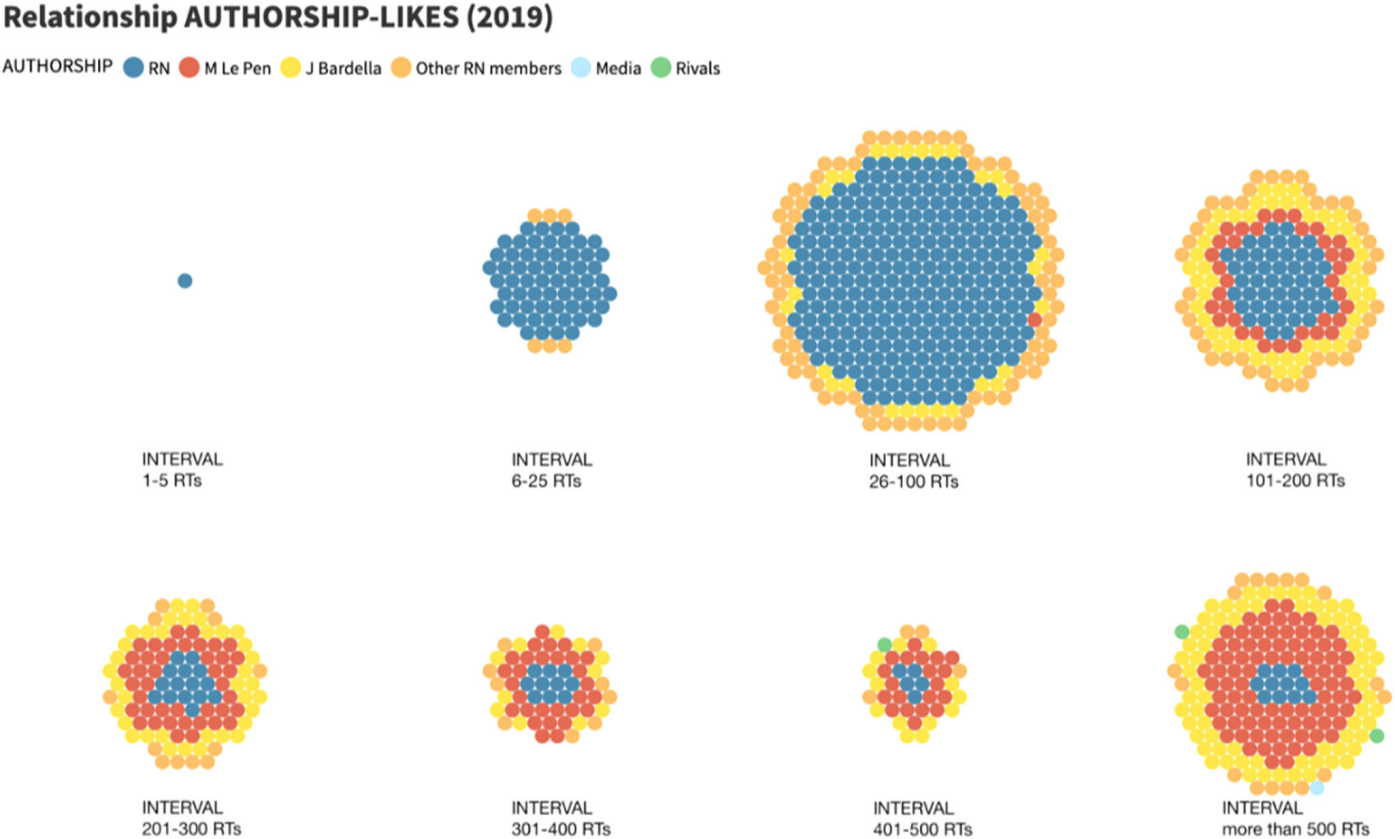
Relationship between RN authorship and range in favourites/Own elaboration.

The involvement of the audience in the form of ‘likes’ shows similar behaviour, but even more defining results. In particular, measuring the number of ‘likes’ in each tweet in relation to its authorship (Fig. 4), the fruitful contribution of new authors to the range that RN publications can achieve is even better appreciated. On the one hand, there is a marked difference in the support received by the tweets from the populist party, which are mainly in lower intervals (1–3), and the retweets from individual politicians, which have increased in quantity and quality of engagement, clearly dominating the higher intervals.

The latest evidence of the RN’s change of strategy is resolved by comparing the written feedback obtained in both election campaigns. First of all, the reduction in the number of publications in the RN’s profile that received no response from the audience, from 57.1% in 2014 to 13.87% in 2019, should be highlighted. This means that the diversity of actors has led to an improvement in audience feedback in approximately 75% of the sample.

**Fig. 5:**
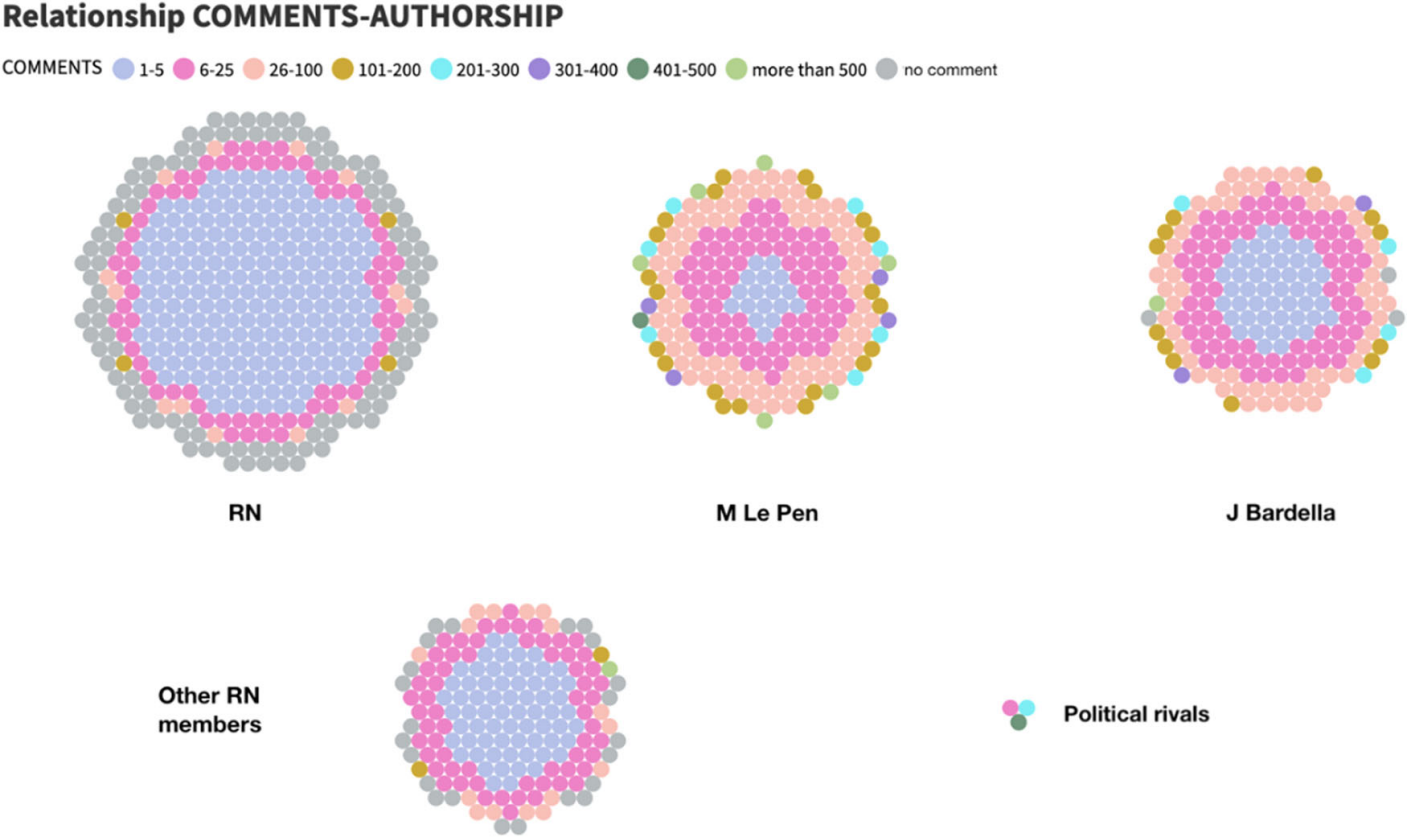
Relationship between range in comments and authorship of tweets/Own elaboration.

In this sense, as can be seen in Fig. 5, we can point to the profile of the populist formation as the author with the highest number of published tweets, but, at the same time, with the lowest degree of response and without reaching any tweet 200 replies. Both Le Pen and Bardella published similar quantitative data, each accounting for around 20% of the total messages collected from the RN account, although the leader’s notoriety eventually led to her messages achieving better results in the higher ranges. Nevertheless, the elaboration of an analysis of the reception of the comments would help to a better understanding of this dynamic, since in this investigation it has not been possible to specify if the feeling of these was positive or negative.

### Hypothesis III: The RN can still be considered a populist party

Having seen the change in communication strategy in a little more detail, we aim at verifying in this third section whether this transformation can lead to a real reform in the character of the RN. Specifically, we question whether opening up to a plural electorate has forced Le Pen to become a party that, like the rest of the traditional forces, once established in the parliamentary arch, from 2014 onwards has supported the prevalence of order among the political class, despite the differences between them all.

However, considering that the new style of communication has only superficially affected the RN’s identity, an attempt has been made to show that the party retains certain characteristics in its communication that make its essence populist. Consequently, the RN’s tweets during both European election campaigns have been analysed to reveal the two features that still link it to an eminently populist nature, as Vallespín and Martínez-Bascuñán (2017) point out:

#### H3.1. The creation of the ‘enemy’

Generally, political enemies are formed in the wake of parliamentary events, except in the case of populisms, whose second lesson—after the appeal to a united ‘people’—is, in fact, the construction of the ‘other’. This enemy is drawn as an entity with multiple faces and various defects, which have led this antagonistic French ‘people’ into the critical situation in which it finds itself. As can be seen, this synthesis of ambiguous tints could include the entire range of enemies of the RN, who could be blamed in any situation.

However, at this point, three nuances should be noted that distinguish the construction of an enemy for the RN: firstly, in this work, it is not expected to nominate and verify who the party’s adversaries are, as no qualitative analysis of the discourse has been made. Secondly, it is also worth recalling the game of representations, and not confrontations, to which Le Pen aspires on social networks. And thirdly, it is necessary to differentiate between the two epithets that draw the RN.

On the one hand, being a populist political party involves the figure of ‘enemy’ implies a disaffection that emanates from both media actors and politicians, whether they are competitors for their vote or antagonistic ideologues. On the other hand, being extremist gives political meaning to the empty words that the populists utter. In the case of the RN, the fact that it is an extreme right-wing party gives to its discourse some conservative doctrinal principles that should act as the motivating reason for its positioning in the right-wing bloc, although its aspirations might be different.

However, while ‘populist’ and ‘extremist’ are by no means mutually exclusive, the RN today prefers to be labelled ‘populist’ rather than ‘ultra-right’. Since her appointment as party leader, Le Pen has tried to impose a regenerative line to break the ties that bind the RN to the extreme right, and consequently to the previous racist and fascist policy practised by his father, Jean-Marie Le Pen. The RN has even gone a step further, advocating to present itself as a political alternative to French representative democracy, wanting to eliminate the right and left factions in order to propose a new classification between globalizers (*mondialistes*) and nationalists (*nationaux*), from which the RN could benefit more by a discourse supported by both progressive and conservative votes.

This change in the RN’s position also changes who the figure of the enemy is. In 2014, when France was immersed in a climate of political discontent, the RN communication strategy was based on creating a multiplicity of diffuse enemies through its “representations” in order to differentiate itself from the traditional actors. This technique led to a reduction of their interaction—positive or negative—with the accounts of the establishment representatives to a minimum. In contrast, in the campaign for the 2019 European elections, it was predicted that the political contest would be decided between Macron’s European subsidiary and RN. Therefore, its strategy was focused on causing a stir in the media to increase its visibility.

**Fig. 6:**
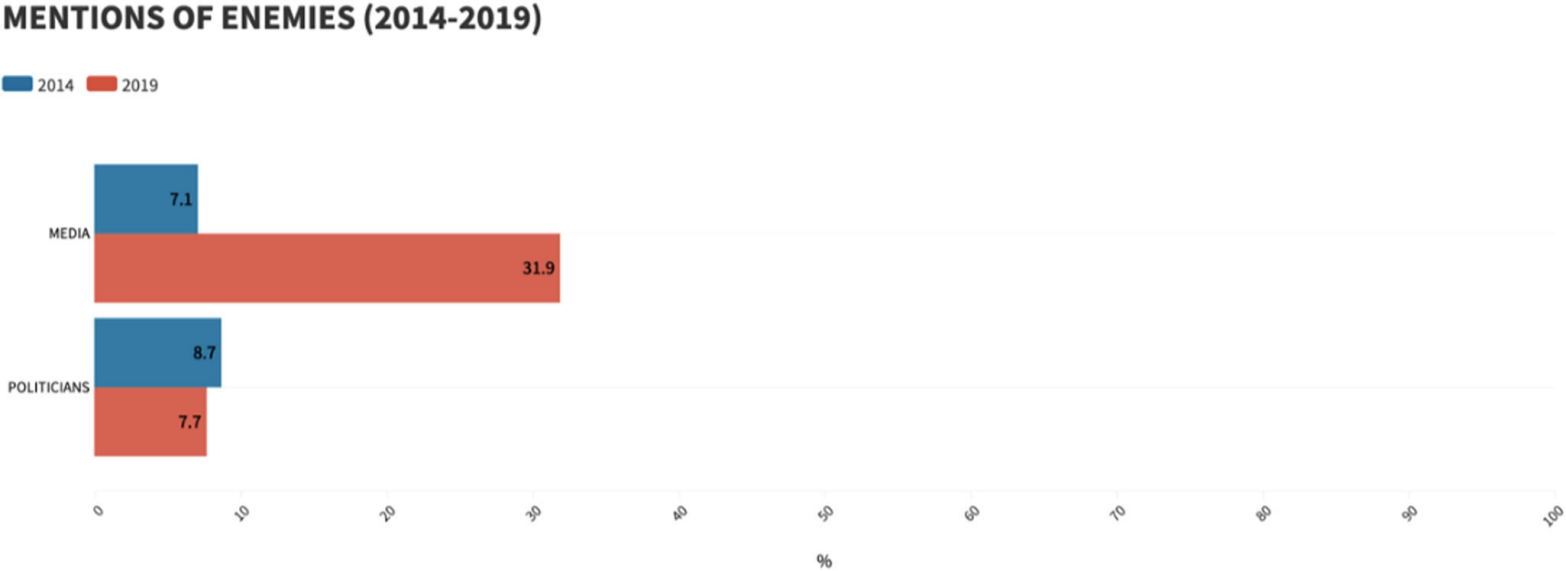
Evolution in the mentions of enemies (2014–2019)/Own elaboration.

In this way, the dynamics relating to mentions carried out in both election campaigns can be understood (Fig. 6). Whereas 5 years ago, confrontation was not sought, therefore, the combined percentage of appeals to political and media actors amounted to barely 15%; in 2019, on the contrary, media attention was sought and therefore they became the most mentioned actor (31.9%), while direct calls to political agents decreased slightly in proportion (7.7%).

Likewise, if we relate these results to the purpose for which they are mentioned, several curious facts can be glimpsed. In 2019, the references to the media stood out in both ways, as the RN aspired to measure itself in a double game with television, radio and the press. On the one hand, it aimed to exert pressure and influence on the thematic agenda. This is why its total number of mentions for complaints (56) is almost ten times the number of reproaches (6) made in 2014. Furthermore, in view of the dichotomous between the candidates Loiseau (Macron) and Bardella (Le Pen), the RN’s strategy in 2019 consisted of positioning the “fourth power” as support for its rival (*Tweet* 1)^1^.
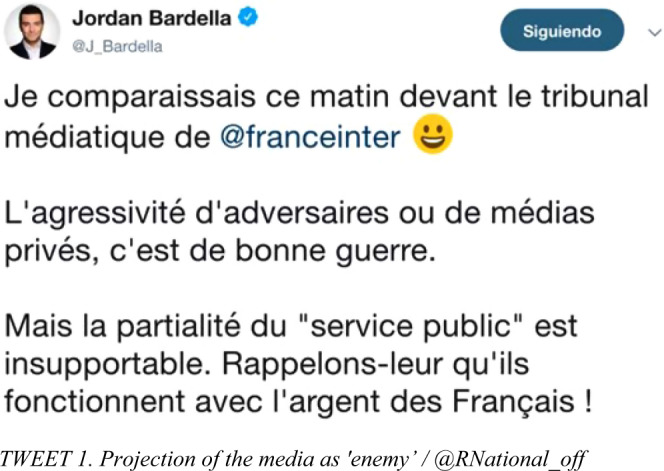


#### H3.2. Polarisation in the use of Twitter

In order to see whether the way in which the RN uses Twitter stimulates nativism, it must first be shown that there is a Manichean double discourse, where good is related to Le Pen’s own party and evil is attributed to the ‘enemy’. To this end, the categories of the ‘purpose’ variable have been divided according to whether they projected a positive or negative tone. Thus, the assertive faction includes the propaganda of meetings, the publicity of its interventions in the media, the mobilisation of the vote and the exaltation of identity (and, in 2014, the electoral programme that the RN disseminated through links to its website). On the contrary, the accusatory position includes confrontation and complaints (and enunciations about its 2019 programme, since they appeared as an excuse for general criticism rather than as the central theme of the tweet).

So, calculating the cumulative percentage of each speech, we can see how, in 2014, the party focused its efforts on a single strategy: the communication of its proposals to its supporters (7/10 publications). However, in these latest elections in 2019, the results showed that the RN combined two strategies in a balanced manner, leading to a reduction in its propaganda behaviour in favour of confrontation.

**Fig. 7:**
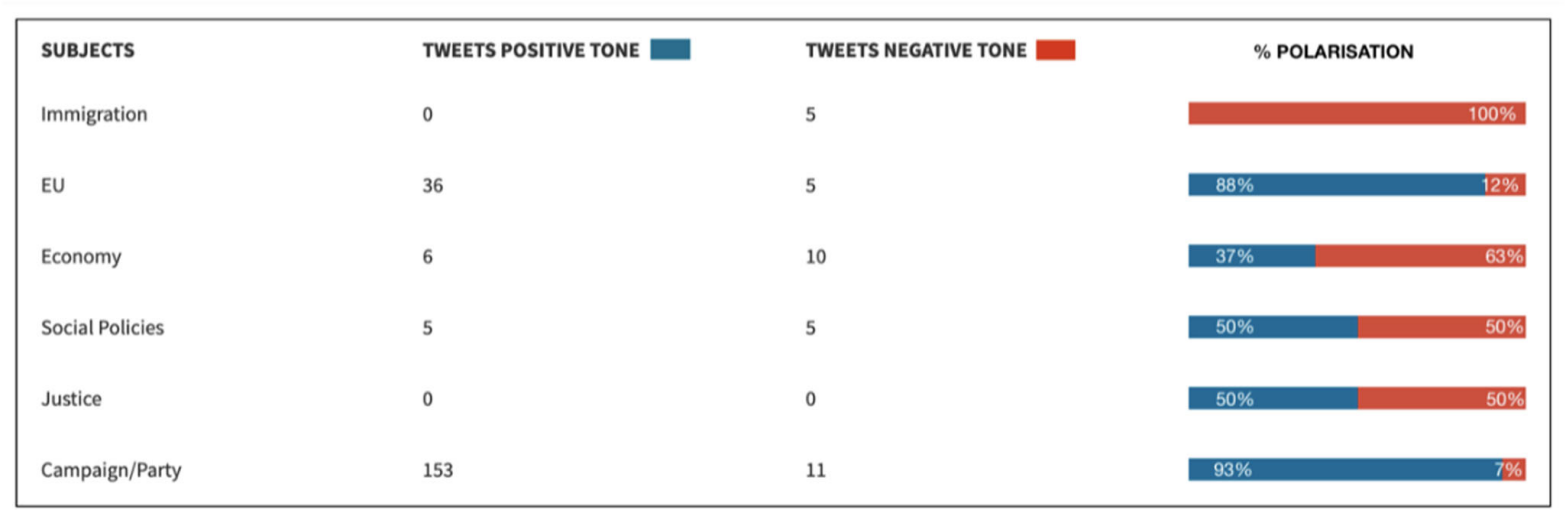
Evaluation of the tone in which each theme is narrated (2014)/Own elaboration.

Therefore, once the double discourse of the NR has been established, the next step was to relate each of the different themes to the favourable or adverse character attributed to it. In this way, following the RN’s predisposition to promote its arguments in its official profile, in Fig. 7,^2^ we can see how, in 2014, posts about the party and the campaign (93%) were spoken about in a positive way, concretely, about France’s exit from the EU (88%). On the negative side, emphasis was placed on the problems of immigration (100%) and the crisis of the euro (63%), as Le Pen’s party was opposed to a common single currency at that time.

**Fig. 8:**
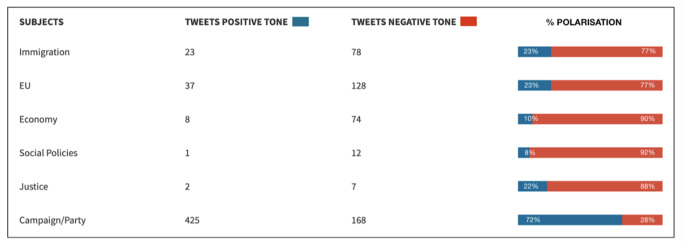
Evaluation of the tone in which each theme is narrated (2019)/Own elaboration.

By contrast, in 2019, there is a clear polarisation of the message, with the party ceasing to promote its ideas on any subject and devoting itself to two very different movements: ‘purifying’ its party and criticising its wide repertoire of enemies. As can be seen in Fig. 8, all the messages written in positive terms refer to his party and the European election campaign (72%); while all the publications on specific subjects have a pessimistic and judgmental tone. In the case of immigration (77% negative), in favour of closing borders; and as for the EU too, 77% of the tweets are critical of the supranational entity because the party is in favour of a Europe of nations with respective sovereignty.

As for the economy, 90% of the messages criticise the liberalism allowed by the French government, since the RN calls for greater protectionism of France’s primary and secondary sectors (agriculture and industry). Along the same lines, it is worth noting the lack of discourse on the pressing issue of climate change, on which the RN resolves the problem by tackling it solely from economic factors such as the promotion of “localism” (sic)^3^ (see Tweet A.1.).

Finally, social policies and discussions on justice issues are intertwined, as all the messages refer to the case of Vincent Lambert, a quadriplegic in a vegetative state since 2008, whose parents oppose the disconnection that his wife and the French and European judicial bodies have endorsed on several occasions. In this sense, between 88% and 92% of the messages show negativity towards judicial decisions and the right to a dignified death.

In short, it has been proven that in the 2014 election campaign, within the RN’s populist nature, the most exploited feature was its propagandistic sense, trying to convince followers to get their vote through utilitarian persuasion, narrating its programme in a positive manner. However, the results of 2019 show that Marine Le Pen’s party used its official Twitter account to polarise its discourse, equating its actions with moral good and those of its enemies with evil in a modelist manner.

## Conclusion

Populisms have proved to be effective in the use of digital tools, managing to win the support of the social audience, mobilising it to the ballot box and translating the retweets into votes. In this sense, the objective of this article was to demonstrate how the RN has attempted to carry out an image whitening in order to increase the potential electorate, and, furthermore, how this good online communication strategy has allowed an extreme right-wing party to maintain its electoral figures, despite the fact that, at the 2019 European elections, it was facing a more complex context. To this end, three initial hypotheses were proposed, for which conclusive evidence has been gathered with which we can confirm what is set out below:

In 2014, the RN had monotonous, unattractive and redundant communication. On the contrary, examining the May 2019 campaign, it can be confirmed that the RN has valued its presence on Twitter as a major resource in its strategy. This implies giving a minimum of control of the conversation to the online community, but without losing the homogeneity of its message. Likewise, more regular communication in networks (254 tweets in 2014—1002 in 2019) also helps to create a ‘spider’s web’ made up of sympathetic users, who are asked to contribute so that, on a horizontal level, they can convince the undecided in their online community.

This feedback led to further propagation of its content, i.e. users not following RN were also exposed to its electoral message increasing their chances of becoming potential voters for the party. This is due to the incorporation of new actors, whose radius of reach has expanded, causing a greater virality of its contents. The only possibility of victory was based on a whitening of his image that would allow RN to reach a cross-section of the public with the same kind of ambiguous and nativist message.

On the exposure level, the online communication strategy (an increase from 8% to 32% in mentions directed at the media) has helped the RN to place itself in the spotlight, giving it greater influence over the communication agents and greater visibility to the audience, through confrontation with the media, which they have labelled as enemies. Therefore, we can affirm that the new image fulfils the expectation of *captatio benevolentiae*, that is, with the politically correct appearance it manages to add up without scaring. Hence the RN once again managed to win over 23% of the French electorate in the European elections of May 2019.

On the political level, however, the RN’s change has not really penetrated its identity. The 100% increase in the specific issues that maintain a negative tone indicates that its discourse has become even more polarised with the aim of creating an enemy to blame (‘elites’), while the positive record is used to encourage the ‘people’ to vote for the RN and fight to regain lost values. In conclusion, we can say that the RN remains a national-populist formation that feeds on confrontation and propaganda. The creation of ‘enemies’ and the moralistic polarisation of their behaviour ratify our third hypothesis: essential characteristics of populism are still to be found in RN publications.

As has been made clear in this research, the power of the word and its framing can give victory to a party that, in many respects (climate change, feminism) lacks an argumentatively sustainable ideology. In this sense, future lines of research that would be appropriate to complete this investigation could start with a review of the discourse (as pointed out in the results and discussion section). Given that Le Pen’s party moves preferably in the game of representations, a study of the theory of signs or semiotics would be ideal to finally approach the topic from both a qualitative perspective and a quantitative one.

## Supplementary information

Tweet A.1.

## Data Availability

All data generated or analysed during this study are public and can be extracted from the corresponding Twitter accounts. Nevertheless, the datasets generated during the current study are available from the corresponding author on reasonable request.

## Footnotes

1 “I appeared this morning before the media tribunal of @franceinter. The aggressiveness of opponents or the private media is admissible, but the partiality of ‘public service’ is unacceptable. We must remind them that they work thanks to the money of the French!”—own translation.

2 The statistical reading of this data is as follows: The % of comments on this subject are positive (red) or negative (blue).

3 ‘Localism’ is a translation of the term ‘*localisme*’ used by Marine Le Pen to speak of protectionism in ecological matters (RN, 11 June 2019).
