# Supplementary figures and images for: Populism, cyberdemocracy and disinformation: analysis of the social media strategies of the French extreme right in the 2014 and 2019 European elections
Source: Humanit Soc Sci Commun. 2023 Jan 18;10(1):23. doi: 10.1057/s41599-023-01507-2 (PMC9848023; doi:10.1057/s41599-023-01507-2)

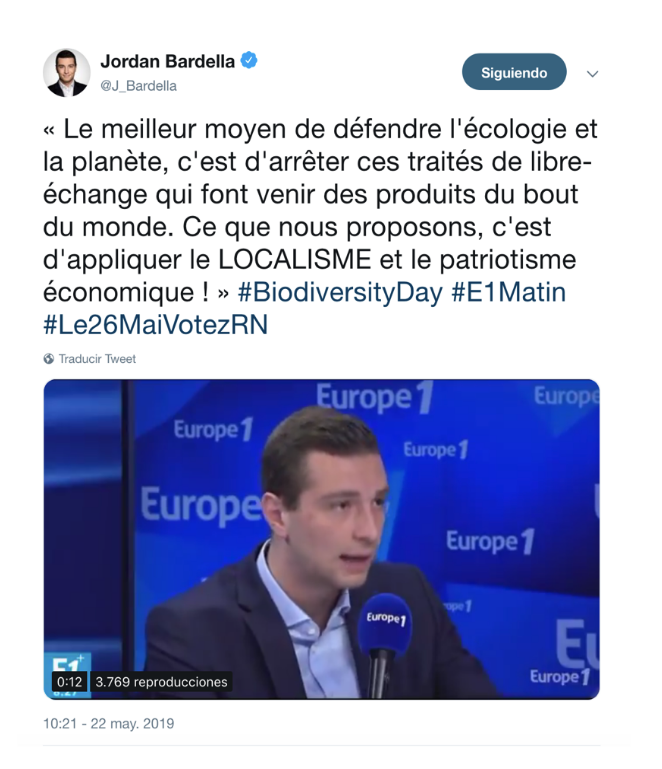

Supplement: Supplementary file 1 — Tweet A.1. [file 41599_2023_1507_MOESM1_ESM.png]
